# Recycled Aggregates from Ceramic and Concrete in Mortar Mixes: A Study of Their Mechanical Properties

**DOI:** 10.3390/ma15248933

**Published:** 2022-12-14

**Authors:** Santiago Rosado, Jorge Costafreda, Domingo Martín, Leticia Presa, Lidia Gullón

**Affiliations:** 1 Fundación Gómez Pardo, 28003 Madrid, Spain; 2 Departamento de Ingeniería Geológica y Minera, Escuela Técnica Superior de Ingenieros de Minas y Energía, Universidad Politécnica de Madrid, 28003 Madrid, Spain; 3 Laboratorio Oficial para Ensayos de Materiales de Construcción, 280906 Getafe, Spain

**Keywords:** ceramics, concrete, mortars, cement, mechanical strength

## Abstract

In recent decades, large amounts of construction and demolition waste (CDW) have been generated and accumulated throughout Europe, which is a challenge to manage and control nowadays. This work shows the results of a study carried out with samples of ceramic recycled aggregates (CRAs) and recycled concrete aggregates (RCAs) mixed with cement (C) in mortars. The main objective of this research is to demonstrate how, by adding CRAs and RCAs to a mixture of cement and natural aggregate (NA), it is possible to develop a high-strength mortar and achieve the best mixing ratio. To achieve these objectives, the characterization of the samples was initially carried out such as XRF, XRD and SEM. Next, tests were carried out on the products obtained, such as the consistency of the fresh mortar and the density of the specimens. Finally, a study of mechanical compressive strength was performed at 7, 28 and 200 days. The results show that although both CRAs and RCAs negatively affect the curing process of the specimens, it is possible to develop mortars with compressive strengths greater than 20 MPa. An obvious increase in mechanical compressive strengths was seen between 7 and 200 days of analysis. The results achieved in this research could be an important guide for the management of CDWs by local industries, thus favouring the development of the circular economy.

## 1. Introduction

In 2018, the consumption of sand and gravel in the European Union (EU) was close to 2.25 billion tonnes, and since 2015 it exceeds 2 billion tons per annum with a tendency to increase. Taking into account that the total consumption of raw materials of all kinds in the EU is close to 7 billion tonnes, it is possible to have an idea of the impact that construction products have on the environment [1]. Up to 40% of all raw materials extracted from the lithosphere are used in this sector, responsible for approximately 50% of global greenhouse gas emissions [2]. Specifically, this sector uses 85% of the natural sand produced in the world [3] and the cement industry alone is responsible for 5–8% of CO_2_ emissions [4,5,6,7,8]. CDW is the material left over from the construction, refurbishment and demolition of roads and buildings. It is a mixture of several different materials including inert waste, non-hazardous waste and hazardous waste. The European Union generates 850–970 million tonnes of CDW per year [9,10]; this figure represents just over 30% of the total waste generated every year [11]. Currently, large quantities of these materials end up in landfills without any form of recovery or reuse [12].

Due to the above, it seems evident that there is a need to shift from a linear economy to a circular economy wherein waste becomes secondary raw material, as is recognised by the EU through the Raw Materials Initiative [13]. The European Green Deal refers to the construction industry as one of the key issues for green transition, and can contribute in a fundamental way to Europe’s carbon neutrality which must be achieved by 2050 [14]. In fact, although the Waste Framework Directive [15] aimed for 70% of CDW to be recycled by 2020, the average recycling rate for this type of waste in Europe is 50%, although in certain countries such as Denmark, Estonia or the Netherlands the rate goes up to 90% [16,17]. Considering that CDWs are made up of materials that can be recovered again in the same sector, the possibilities of reducing the consumption of raw materials in construction are enormous.

Concrete is the most widely used building material in the world, so recycled aggregates from concrete are highly abundant [18,19]. These differ from natural aggregates in that they are formed by two components, namely the original aggregate and the cement adhered to it. The specific characteristics depend on the production process used in the waste management plant and the origin of the material [20,21].

Despite the importance of concrete, mortars are a great alternative to include recycled aggregates due to their usual use as non-structural material. In fact, this alternative has been closely studied for years [22,23]. Although, in terms of mechanical strength, better values are obtained using natural materials than with recycled concrete, the strengths are still good enough to be used [16]. Even in low replacement ratios, some properties improve the natural material [24]. In addition, in places with limited access to quality natural material, this can be a better alternative to traditional ones [22].

The case of ceramic waste is similar to that of concrete, with the advantage that ceramic aggregates have a pozzolanic activity that increases with the decrease in particle size, increasing by up to 40% the proportion of substitution by natural aggregates without significant differences in the properties of the fresh and hardened mortar.

The objective of this study is to demonstrate how construction and demolition waste as common as crushed concrete [24,25,26] or ceramic from tiles and bricks [27,28,29] can be used as a secondary raw material that replaces natural aggregates, thus reducing the impact of the mining activity.

With this type of action, it is possible to transform what was initially considered waste into a secondary raw material, which is reincorporated into the market, thus extending its life cycle. This greatly reduces the amount of discharge that takes place, in addition to optimizing the consumption of natural resources.

Furthermore, this research positively contributes to the Sustainable Development Goals (SDGs), directly so to SDG No. 12 (responsible consumption and production) and No. 13 (climate action), as well as, in a more indirect way, to No. 9 (industry, innovation and infrastructure), No. 11 (sustainable cities and communities) and No. 15 (life on land). This research promotes new value chains in the construction industry, as well as the recycling of construction waste present in cities and the recovery of damaged spaces.

## 2. Materials and Methods

### 2.1. Materials

The following is a description and identification of the origins of the material used. Three different types of waste have been used, as well as a cement and an aggregate of natural origin.

The ceramic waste comes from an old brick factory located in San Fernando de Henares (Madrid, Spain). These residues consisted of fragments of variable sizes (5 to 40 cm) from tiles, bricks and structural parts. These broken pieces had a rather flaky appearance and ranged in colour from bright orange to dull red.

The crushed concrete waste came from a construction and demolition waste treatment plant located in Madrid. It resembled river sand but with a lighter colour. For the study, 3 tonnes were separated and homogenized in order to have a representative sample.

The ceramic waste was prepared in the laboratory by crushing it to a size of less than 4 mm in a jaw crusher to obtain a ceramic recycled aggregate (CRA). The concrete waste was treated at source to obtain a recycled concrete aggregate (RCA) of less than 4 mm.

Natural fine aggregates (NAs) were supplied by the same manufacturer as cement. In this case, it was a 0/6 fraction of natural origin (river sand).

**Figure 1:**
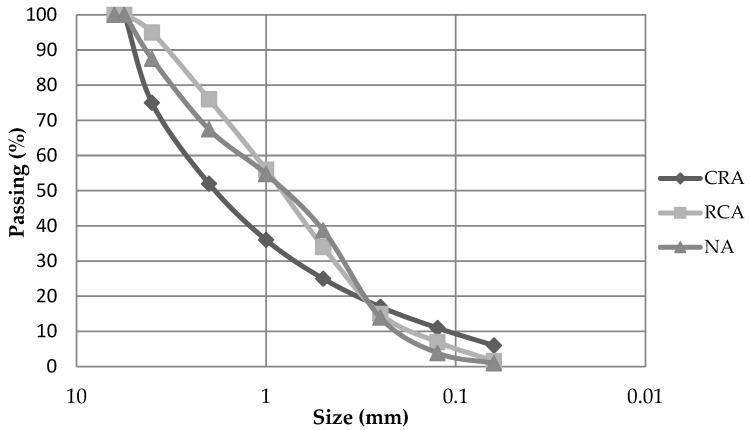
Particle size distribution for ceramic recycled aggregate (CRA), recycled concrete aggregate (RCA) and natural aggregate (NA) samples.

Figure 1 shows the particle size distribution of the materials. Although the maximum aggregate size in mortars is 8 mm, maximum sizes of 4 or 6 mm are normally used to have a more uniform product.

The cement (C) used for the tests is a CEM II/A-M (V-L) 42.5 R with a content between 12 and 20% of siliceous fly ash and limestone, with a nominal resistance at 28 days of 42.5 MPa [30].

The water used for the tests was deionized type II [31].

### 2.2. Methods

For the characterization of the samples, dispersive wave X-ray fluorescence (XRF) tests were performed using a BRUKER S4 PIONEER (Bruker, Billerica, MA, USA) instrument with a detection limit of 0.01% for oxides of Al, Ca, Fe, K, Mg, Na, S and Si.

To establish the mineral species, a Bruker X-ray diffractometer (XRD) model D8 ADVANCE (Bruker, Billerica, MA, USA) was used. The angular interval used was between 2 and 65° with a step size of 0.02° and a time of 1 s. All samples were analysed: natural sand, both recycled aggregates and cement. In the case of the final mortars, only REF-I and M-VI mixtures were analysed in order to study the potential differences between the most different mixtures performed.

The mineral analysis of the samples was performed using a Scanning Electron Microscopy with Dispersive Energy Detector (SEM−EDX) model JEOL JSM 820 (Jeol, Japan). The preparation of samples consisted of grinding below 63 microns after which the samples were coated with gold to make them conductive.

The grinding equipment is a Siebtechnick Scheibenschwingmühle TS 1000 ring mill (Siebtechnick, Germany).

The particle size tests were performed according to Standard EN 933–1 [32]. Sieving was performed dry. The sieve openings were 0.063 mm, 0.125 mm, 0.25 mm, 0.5 mm, 1 mm, 2 mm, 4 mm, 5.6 mm and 6.3 mm. The diameter of the used sieve was 200 mm, made of stainless steel. The tests were carried out by hand without shaking equipment.

Both the manufacture of specimens and the determination of compressive strengths were carried out according to Standard EN 196−1 [30]. The strengths were determined at 7, 28 and 200 days.

Several mixtures were developed to obtain a mortar with a high amount of recycled material and with competitive properties compared to a natural one. In the design of these mixtures, the mortar used as a reference was the one used in the standard for assessment of cement resistances [30]: 25% cement and 75% aggregate. Dosages are shown in Table 1.

Two reference samples (REF-I and REF-II) were manufactured: the first with natural materials and the second with an addition of 5% (cement) of ground concrete below 70 microns, a proportion which has yielded good results [33,34]. Study mixtures (M) were identified in a correlative manner (I, II… VI) replacing various proportions of natural aggregate with recycled concrete aggregates (RCAs) and ceramic recycled aggregates (CRAs) as follows:

- M-I: 50% RCA;
- M-II: 50% CRA;
- M-III: 50% RCA and 50% CRA;
- M-IV: 60% RCA and 40% CRA;
- M-V: 42.5% RCA and 42.5% CRA;
- M-VI: 35% RCA and 35% CRA.

The first two references (REF-I and REF-II) were made with natural aggregate (sand). The mixtures M-I and M-II were made to separately evaluate the behaviour of the residues. Mixtures M-III, M-V and M-VI contain equal parts of residue and different natural sand contents. Finally, the M-IV contains a total replacement of sand with recycled material but with a greater amount of concrete due to the greater water absorption capacity of the ceramic recycled aggregates.

The consistency study of the mixtures was carried out using the procedures indicated in Standard EN 1015–3: 2000 [35]. The reference value was set at 175 ± 10 mm. The amount of water used in the mixtures was adjusted to obtain these consistency values. However, in the case of the reference samples, the amount of water used was the indicated by the standard [30].

The wet density of the specimens was calculated by adapting the Standard EN 1015–10 [36]. The wet mass of the test specimens was determined 72 h after its preparation. The volume was calculated theoretically based on the dimensions of the moulds used. Although there is a deviation from how to determine the density of dry mortar, the purpose was not to obtain this value but to monitor the quality of the mixtures with respect to the development of the strengths.

## 3. Results and Discussion

Regarding the sample composition by XRF of ceramic recycled aggregates (Table 2), it is important to highlight the high proportion of both alumina and silica, surpassing the values of other studies [3,37] and even those of residues from the same study area [16]. This was similar to the recycled concrete aggregates where the silicon content stands out positively, probably motivated by the siliceous origin of the aggregates of the original concrete and superior to other recycled aggregates in the region [16]. The low amounts of sulphates in the recycled aggregates should be highlighted because a high presence of this compound is related with a decrease in the durability of the final products. This issue has a special importance in the case of recycled aggregates where a contamination by gypsum is common due to a bad separation process in origin.

**Figure 2:**
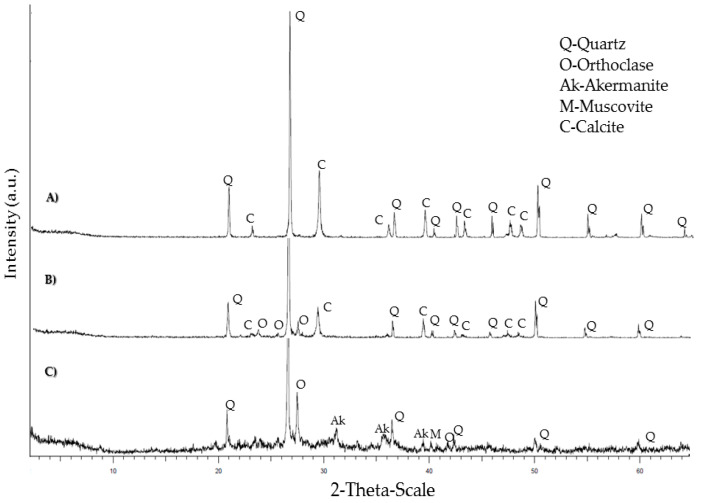
XRD mineral analysis of sands (**A**), concrete (**B**) and ceramics (**C**).

**Figure 3:**
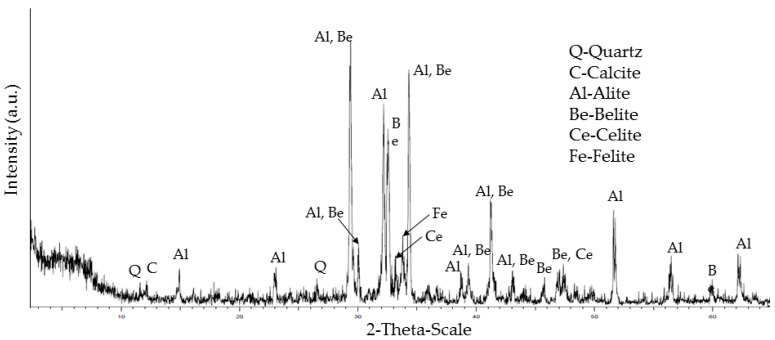
XRD mineral analysis of cement.

X-ray diffraction mineral analysis shows the classical mineral present in ceramics, concrete, natural aggregate and cement (Figure 2 and Figure 3). Both concrete and natural aggregate show quartz and calcite in their composition. In the case of ceramics, the presence of quartz is observed in addition to phyllosilicates, potassium feldspar and plagioclase. This agrees with the XRF analysis, showing a high presence of silica and alumina. In the case of the cement XRD analysis, the typical cement minerals are shown: alite, belite, celite and felite. The peaks of calcite show the presence of the limestone addition while the fly ashes addition is related with the quartz.

**Figure 4:**
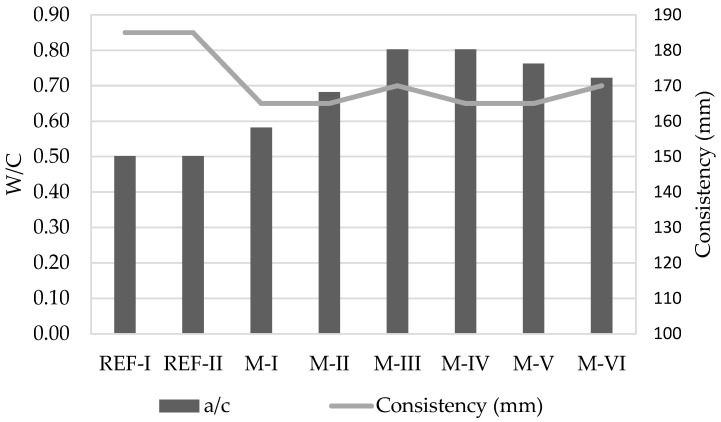
Water/cement ratio (*w*/*c*) and consistency of all mixtures.

Figure 4 shows the water consumption of the mixtures as a function of the water/cement ration (*w*/*c*). The value established by standard [30] of 0.5 as *w*/*c* ratio was taken as a reference (REF-I and REF-II).

Upon observation, all mixtures have a higher water consumption (0.58–0.80) for a similar consistency (165–170 mm) because in general, it is accepted that the use of recycled aggregates implies a greater amount of water [38] than when these come from lighter concrete [39,40] and considerably higher for those of ceramic origin [41]. This elevated water consumption is mainly due to the higher porosity and the higher surface area of the recycled aggregates than natural ones. The cement paste has a high porosity and it is presently adhered to the recycled aggregates. The crushing processes manufacture recycled aggregates with a greater angularity than natural ones; therefore, the recycled particles have a higher surface for the same volume. In fact, observation shows that the M-I mixture requires a higher proportion (0.58), whereas in M-II it is even higher (0.68). M-III and M-IV have a higher consumption (0.80) than M-I and M-II because they do not have natural aggregates. A similar but less noticeable case occurs in M-V (0.76) and M-VI (0.72), where the amount of recycled aggregate progressively decreases.

**Figure 5:**
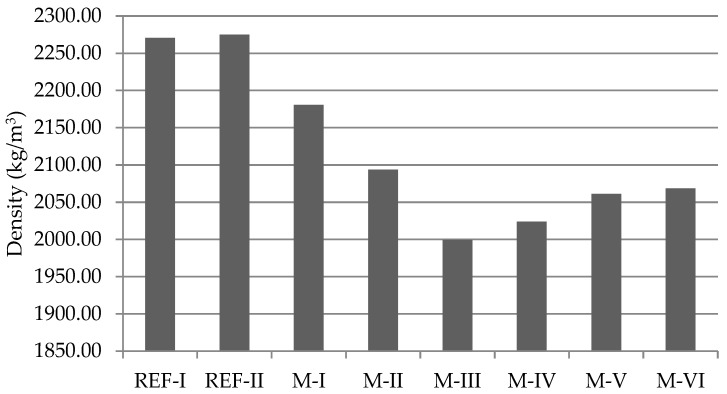
Density of mortar specimens.

The density of the hardened mortar mixes is highly affected by the recycled aggregates, as it is shown in Figure 5. The use of recycled aggregates causes a decrease in the density of the final mortars.

The density of recycled aggregates is typically lower than that of natural aggregates [38,41] (1.22 g/cm^3^ for NA compared to 1.59 g/cm^3^ for recycled concrete aggregates [38] and 1.14 g/cm^3^ for NA compared to 1.04 g/cm^3^ for ceramic recycled aggregates [41]), which has a direct impact on the density of the final specimens (Figure 4). In this case, the lightness of recycled ceramic aggregates causes mortar mixtures with a high content of this to be less dense than those with recycled concrete aggregate content. There is some parallelism between high water absorption and low density, common in recycled aggregates [38].

**Figure 6:**
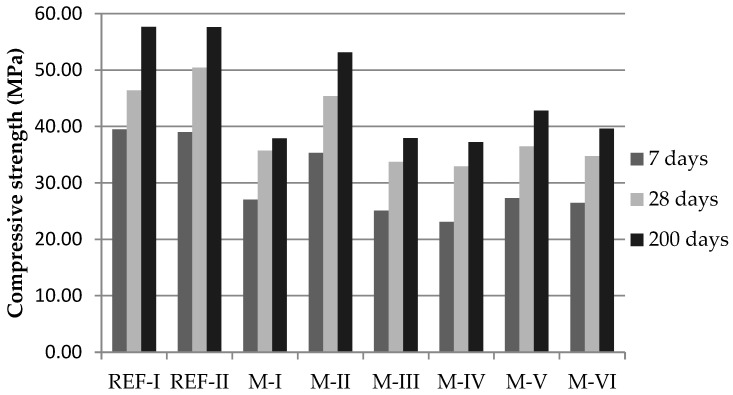
Compressive strength of the mortar mixes.

As expected, a decrease in the proportion of recycled material (M-V and M-VI) provides slightly higher mechanical strength (Figure 6). At 28 days, values of 35.5 and 34.8 MPa are obtained, respectively. Compared to those obtained by completely substituting M-III and M-IV: 33.8 and 32.9 MPa. Nevertheless, these differences are not enough to justify the use of natural material.

The strength increases as the curing time increases. This effect is much more pronounced in M-II (17.8 MPa) than in M-I (10.9 MPa) due to the pozzolanic nature of ceramic materials such as those used in this research [37].

In the reference values, it should be noted that the addition of ground concrete to cement does not have any negative effects. Comparatively, ceramic recycled aggregates behave more efficiently than recycled concrete aggregates. However, the results of M-IV, which includes a higher proportion of recycled concrete aggregates than ceramic recycled aggregates, are not very different from those obtained in M-III where the proportions are equal.

In relative terms of compressive strength, the most suitable mixture is M-III (Figure 6), as it completely uses recycled aggregate instead of natural aggregate (50% RCA and 50% CRA). Despite the decrease in strength, values of 30 MPa are reached, which is superior to high-quality mortars used in construction (10 MPa).

It is interesting to look at the results of the mineral analysis by XRD for the REF-I and the M-VI mixtures since they are the most different samples from each other.

**Figure 7:**
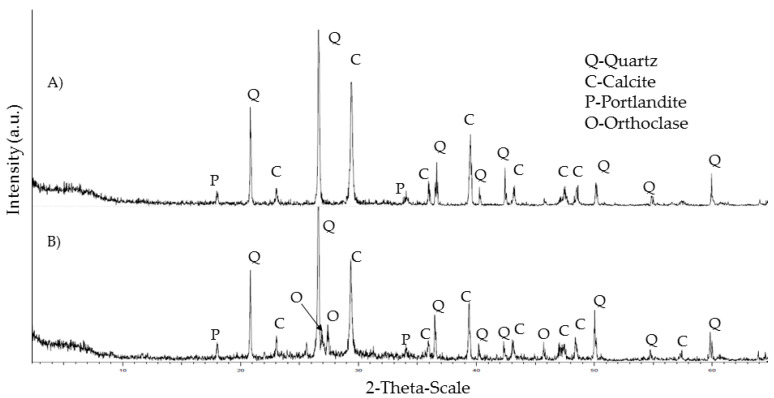
Mineral analysis of mortar specimens REF-I (**A**) and M-VI (**B**).

**Figure 8:**
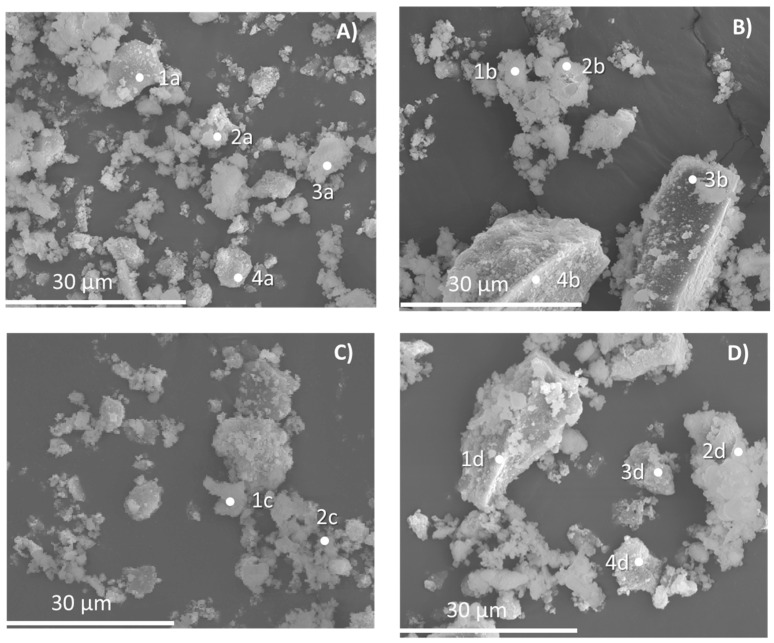
Microphotographs obtained from scanning electron microscopy. (**A**) REF-I, (**B**) M-I, (**C**) M-II and (**D**) M-VI.

In the case of REF-I (A), the minerals of mortar (calcite and portlandite) as well as quartz from the siliceous aggregate are observed (Figure 7), whereas in M-VI (B), potassium feldspar is also observed from the addition of recycled ceramic aggregate instead of siliceous aggregate. This feldspar contains typical elements from ceramic materials such as aluminium, potassium, calcium and magnesium in the form of silicates and aluminates of calcium, potassium and magnesium. The presence of these materials is the main difference with the REF-I mixture. The alite typical from the cement is not shown in the figure, although its presence is confirmed by the SEM analysis. This is due to the fact that the main peak is covered by the calcite peak (29–30) and it is possible to see an accumulation of signal in 32–33° of the other main peaks of this mineral. The presence of the hydrated phases of the cement such as calcium silicates (22–23°) or aluminates (18°) are covered by the presence of other main minerals, such as quartz and calcite. The portlandite is shown in the figure. The microphotographs obtained by scanning electron microscopy are shown in Figure 8A–D.

In the microphotographs of the mortar samples, it is possible to see massive aggregates of sizes less than 10 µm, which correspond to alite (1a, 2a, 1c, 2c and 3d). In the sample REF-I, the presence of these aggregates is very high and is found together with some crystals of calcium oxides (3a and 4a). However, the presence of these mineral phases in the samples containing different substitutions is significantly lower. The M-I sample has large quartz crystals (3b and 4b), as expected when substituting part of the natural aggregates for recycled concrete aggregates. On the other hand, the M-II sample, in which the aggregate was replaced by ceramic recycled aggregates, presents a morphology similar to that of the reference cement (REF-I), formed by small clusters of massive aggregates. However, in its composition, significant contents of potassium and aluminium, common elements in ceramic materials, are observed. Finally, it is noted that the M-VI sample presents a more complex morphology. Large quartz crystals are observed (1d), similar to the M-I sample, but the presence of massive aggregates is greater. This sample contains substitutions of both ceramic recycled aggregates and recycled concrete aggregates, which is consistent with the presence of silicates and aluminates of calcium, potassium and magnesium (2d, 3d and 4d) found in the sample.

## 4. Conclusions

First, it is possible to see how recycled raw materials are of high quality in terms of their chemical make-up, because they have a low sulphate content, higher in recycled concrete aggregates, probably due to gypsum contamination. However, the silica content is high (above 50%), a positive aspect in structural terms, although it does not attain the value of the natural aggregate sample due to its siliceous origin.

In physical terms, it is possible to establish two parameters of interest where the relationship between w/c and resistance has been determined.

Recycled materials have a high water consumption which implies a decrease in mechanical strength (higher ratio *w*/*c*). However, in practice, this is not a decisive factor because M-I has a lower water consumption for the same proportion of recycled materials as M-II, which has a significantly higher resistance. Therefore, it is preferable to increase the *w*/*c* ratio in order to obtain fluid and easily workable mixtures than in the ones that are drier and where imperfections are created in the final products, which will affect the resistance to a much greater extent.

On the other hand, even though mixtures with a high content of recycled materials have a significantly lower resistance than those with large amounts of natural material, it is proven that it is technically feasible to develop mortars with proportions of recycled material that are greater than 75%. The mechanical compressive strength values attained are several times higher than the mortars currently marketed (10 MPa). It is likely that in future research it will be possible to achieve values above 80% of recycled material.

These high values prove that it is possible for the construction sector to contribute positively to improving the current consumption of raw materials. Additionally, it has been proven that construction waste has a high-quality recycling route, which can minimize the dumping of this type of waste.

## Figures and Tables

**Table 1:** Mortar mixtures. C—cement, NA—natural aggregate, RCAs—recycled concrete aggregates, CRAs—recycled ceramic aggregates.

	REF-I	REF-II	M-I	M-II	M-III	M-IV	M-V	M-VI
C (%)	25.00	23.75	23.75	23.75	23.75	23.75	23.75	23.75
RCA milled (%)		1.25	1.25	1.25	1.25	1.25	1.25	1.25
NA (%)	75.00	75.00	37.50	37.50			11.25	22.50
RCA (%)			37.50		37.50	45.00	31.88	26.25
CRA (%)				37.50	37.50	30.00	31.88	26.25

**Table 2:** XRF of used materials. C—cement, NA—natural aggregate, RCAs—recycled concrete aggregates, CRAs—recycled ceramic aggregates.

	CRA	RCA	NA	C
Al_2_O_3_ (%)	19.3	4.25	1.55	5.09
CaO (%)	1.77	10.85	0.16	>60
Fe_2_O_3_ (%)	6.68	1.94	0.61	2.70
K_2_O (%)	4.52	1.46	0.61	0.96
MgO (%)	5.69	0.45	0.09	1.80
Na_2_O (%)	1.03	0.54	0.19	0.14
SO_3_ (%)	0.06	0.32	0.02	2.95
SiO_2_ (%)	59.32	72.25	95.44	19.84
LOI (%)	1.3	7.91	14.84	6.45

## Data Availability

The data presented in this study are available on request from the corresponding author.
